# Influence of Risk Factors on Exercise Tolerance in Patients after Myocardial Infarction—Early Cardiac Rehabilitation in Poland

**DOI:** 10.3390/jcm11195597

**Published:** 2022-09-23

**Authors:** Aleksandra Bryndal, Sebastian Glowinski, Agnieszka Grochulska

**Affiliations:** 1 Department of Physiotherapy, Institute of Health Sciences, Slupsk Pomeranian University, 76200 Slupsk, Poland; 2 State Higher School of Vocational Education in Koszalin, 75-582 Koszalin, Poland

**Keywords:** cardiac rehabilitation, myocardial infarction, risk factors, overweight, smoking, diabetes, hyperlipidaemia, hypertension

## Abstract

(1) Background: Prognosis in patients with cardiovascular diseases is significantly influenced by lifestyle and the control of risk factors. Patients after myocardial infarction require special care and promptly introduced cardiac rehabilitation. The primary aim of this study was to identify risk factors and their influence on exercise tolerance before and after cardiac rehabilitation (CR) provided under the Coordinated Specialist Care Programme—Infarct (CSC-Infarct). (2) Methods: The study was carried out at the Cardiac Rehabilitation Centre of Slupsk Specialist Hospital on a group of 112 patients aged 35–87 (62.78 ± 10.09 years) after myocardial infarction (MI), participating in CSC-Infarct. An exercise test (treadmill ECG test), the 6 min walk test (6MWT), echocardiography, blood test (total cholesterol, HDL, LDL, TG), measurement of diastolic pressure ratio (DPr), waist-to-hip ratio (WHR), and BMI were performed in participants on the first and last day of CR. Rating of perceived exertion was assessed with Borg’s scale. (3) Results: The overweight variable had the strongest effect on the increased value of initial: HR rest, HR max, and HR 1 min after exercise compared to subjects with normal BMI. DPr values before and after CR were also higher in overweight patients. Scores of 6MWT were higher in smokers compared to non-smokers. The final MET value was significantly higher in non-diabetic subjects. Hyperlipidaemia was associated with a higher initial HR max and initial HR 1 min after exercise. DPr before CR was also higher. The initial and final MET values were lower in hypertensive patients. Borg’s rating of perceived exertion measured after the final exercise test was also higher in hypertensive patients. Hypertension influenced the initial and final 6MWT scores, which were significantly higher in normotensive patients. (4) Conclusions: CR within CSC-infarction in patients after myocardial infarction improves exercise tolerance. Exercise tolerance in post-MI patients with concomitant risk factors is lower compared to post-MI patients without risk factors.

## 1. Introduction

Cardiovascular diseases (CVDs) cause 47% of all deaths in Europe and 40% in the European Union (EU) [1]. A similar trend is observed in Poland, where mortality due to CVDs is close to 46% and is on average 8% higher than in other EU member states [1,2]. CVDs are a serious health problem as the leading cause of premature death in people under the age of 65 [3,4].

Prognosis in patients with cardiovascular disease is significantly influenced by lifestyle and the control of risk factors. Recently, the Polish, European and American societies for cardiology published updated guidelines on the prevention of cardiovascular diseases [5]. Experts of the European Society of Cardiology have indicated the need to refer all patients after an acute coronary incident to centers offering a coordinated cardiac rehabilitation program, since it is associated with an improved prognosis [6,7].

Studies conducted in recent years revealed that the control of risk factors in the Polish population, although it is gradually improving, is still unsatisfactory [8]. In Poland in order to reduce the number of fatalities and disabilities due to heart failure and to facilitate the return of myocardial infarction (MI) patients to physical activity, the Coordinated Specialist Care Programme—Infract (CSC-Infarct) was introduced in 2017 [9]. CSC-Infarct was the first Polish system of coordinated care for patients after MI. Nine potentially modifiable environmental factors are responsible for over 90% of the risks associated with myocardial infarction, including smoking, obesity, hypertension, hyperlipidaemia and diabetes [10,11].

Smoking is one of the strongest risk factors for cardiovascular disease, including acute myocardial infarction [12]. Obesity has consistently been associated with an increased risk for cardiovascular disease [13]. The mechanisms of obesity and its relation to cardiovascular risks, describing the available treatment options to manage this condition, were described by Cercato and Fonseca [14]. Arterial chronic hypertension is an important risk factor for heart failure, myocardial infarction, and cardiovascular-related death [15,16,17].

The primary aim of this study was to identify risk factors and their influence on test outcomes in a group of patients participating in the CSC-Infarct program. The following hypotheses were made:

- The participation of patients after myocardial infarction in the CSC-Infarct program changes the parameters of exercise tolerance by the cardiovascular system;
- The risk factors determine the obtained parameters of exercise tolerance before commencement and after the completion of the coordinated cardiac rehabilitation program.

## 2. Materials and Methods

### 2.1. Participants

The study was carried out at the Cardiac Rehabilitation Centre of Slupsk Specialist Hospital. We researched between April 2019 and May 2020 on a group of 112 patients aged 35–87 (62.8 ± 10.1 years) after MI, participating in the Coordinated Specialist Care Programme-Infarct (CSC-Infarct). The study group consisted of men (69.6%) and women (30.4%).

### 2.2. Selection Criteria

The criteria for inclusion in the research and exclusion from the research were applied. The criteria for inclusion were: previous myocardial infarction after full revascularization, clinically and haemodynamically stable, without significant arrhythmias, age over 18, systematic attendance at cardiac rehabilitation and informed written consent of the patient to participate in the study. The exclusion criteria were: recent MI (according to the recommendations of the American Heart Association—the first 2 days), unstable angina, stenosis of the left coronary artery, symptomatic severe stenosis of the aortic opening, decompensated heart failure, acute pulmonary embolism or pulmonary infarction, deep vein thrombosis, mobile or fresh thrombus in the heart cavities, myocarditis, endocarditis or pericarditis, aortic dissection, symptomatic second and third-degree atrioventricular block without pacemaker protection (acquired), poorly controlled arterial hypertension, recent stroke or cerebral ischemia, other acute or decompensated non-cardiac disease that may interfere with exercise test performance or worsen during exercise, age under 18 y, and lack of informed consent of the patient to participate in the study.

### 2.3. Instruments

In this study, we analyzed and selected training methods by carrying out exercise tests. An exercise test (treadmill ECG test), the 6 min walk test (6MWT), echocardiography, blood test (total cholesterol (TC), high-density lipoproteins (HDL), low-density lipoproteins (LDL), and triglycerides (TG)), measurement of diastolic pressure ratio (DPr), and BMI were performed in participants on the first and last day of CR. Body mass index (BMI) was calculated from patients’ body weight and height. The level of perceived exertion was assessed with the Borg’s rating of perceived exertion scale. All patients were examined twice, on admission to the day cardiac rehabilitation center and after the completion of the four-week rehabilitation program [18].

Before starting the treadmill exercise test, the patient was examined (clinical history, ECG, blood pressure measurement, resting heart rate (HR)) in order to detect possible contraindications to the test. During the examination, electrodes were placed on the patient’s body in order to obtain an ECG recording. The ECG was constantly monitored and recorded as a record on the computer monitor. Exercise on the treadmill was performed according to the Bruce loading protocol, in which every 3 min (consecutive steps in the protocol), the speed of the treadmill and its inclination increased, and thus the metabolic equivalent (MET) was changed every 3 min, blood pressure was measured and HR. ECG, blood pressure, and HR parameters were also recorded after the end of the exercise, during the rest phase at 1, 3, 6, and 9 min. For our analysis, we used MET; HR: resting HR (HR rest) and maximum HR (HR max) (beats/min), and HR 1 min after exercise (HR 1 min). The criteria for ending the test were fatigue declared by the patient without signs of heart failure, ST depression > 2 mm, detection of new segmental contractility disorders, arrhythmias, increase in blood pressure > 240/110 mm Hg, and achievement of the target HR. Each of these reactions was considered a physiological hypotensive response [18,19].

The 6MWT was performed in a 30 m long corridor, marked every 3 m. A stopwatch and a sphygmomanometer were used during the test. Before starting the test, the patient sat at rest for 10 min. Patients were also instructed not to exercise vigorously 2 h prior to the start of the test. Patients were advised to walk at their own pace and were permitted to slow down or stop as necessary. The aim of the test was to cover the longest possible distance in 6 min, as established in the guidelines of the American Thoracic Society [20]. All patients completed the 6MWT. There were no clinical complications during the tests or in the 5 h after its completion.

Another test performed twice was echocardiography using the Acuson 128 apparatus with a 3.5 MHz ultrasound transducer probe. This test assessed the left ventricular ejection fraction (LVEF/EF) using the Simpson method in accordance with the current recommendations of the American Society of Echocardiography. The assessment was based on mean measurements for three cardiac cycles [21].

Standard blood lipid lipoprotein tests were used to measure the levels of TC, HDL, LDL, and TG.

BMI was calculated from the formula: BMI = weight (kg) ÷ height^2^ (meters). Based on the BMI value, the patients were classified into categories: underweight (under 18.5), normal weight (18.5–24.9), pre-obesity (25.0–29.0), or obesity (above 30.0) [22].

The body fat distribution was calculated based on the waist-to-hip ratio (WHR). The waist circumferences <94 cm (men) or <80 cm (women) were considered normal [23].

The DPr was defined as the average Pd/Pa over the entire diastole, where Pd is the ratio of resting distal coronary pressure, and Pa is aortic pressure [24]. The level of perceived exertion was measured using the Borg rating of perceived exertion scale, which is a subjective estimate of the work intensity undertaken. On this scale, the level of exertion is rated from 6 to 20, where 7 is extremely light exertion, 9—very light, 11—light, 13—somewhat hard, 15—hard, 17—very hard, 19—extremely hard, and 20—maximal exertion [25].

Patients were also interviewed about their comorbidities and risk factors, such as diabetes and smoking, including e-cigarettes or vaping (daily smokers or smokers who have stopped smoking for the last 6 months).

The research was conducted in accordance with ethical principles (see below for further details). The procedure and purpose of the study were explained to each patient, and they provided their written informed consent to participate in the study. Each patient also consented to the use and processing of their medical data.

### 2.4. Procedure

The patients underwent cardiac rehabilitation under the Coordinated Specialist Care Programme—Infarct (CSC-Infarct). Patients entered cardiac rehabilitation under the CSC-Infarct on average 10 days (±1; range 7–13) after complete revascularization of the coronary vessels. Early outpatient post-hospital cardiac rehabilitation under CSC-Infarct lasted for 20 days (4 weeks, 5 days of rehabilitation followed by 2 days of rest). This duration of early cardiac rehabilitation in Poland is financed by the National Health Fund.

All patients qualified for the rehabilitation program received pharmacotherapy in accordance with the standards of the European Society of Cardiology and did not require modification during early post-hospital cardiac rehabilitation.

According to the recommendations of the Polish Society of Cardiology, patients were initially assessed for their functional capacity based on the result of the treadmill exercise test, the 6MWT, and the risk of cardiovascular events, and then prescribed one of the rehabilitation models: A, B, C, or D (Table 1) [9].

### 2.5. Statistical Analysis

All statistical calculations were performed using the methodology and STATISTICA package version 13.0 from StatSoft Inc. and Python open-source programming language [26,27,28]. For quantitative variables, we calculated the mean, standard deviation (SD), median, minimum and maximum values (range), and 95% CI (confidence interval). Qualitative variables were presented using cardinality statistics and percentage values (percentage). The Shapiro–Wilk test was used to verify the normal distribution of quantitative variables. However, the Leven (Brown–Forsythe) test was used to verify the hypothesis about the equality of variances [29,30]. The significance of the differences between the two groups (unrelated variable model) was tested with Student’s *t*-test, Welch’s test (when variance was heterogeneous), or 5 Mann–Whitney’s U test (when conditions for the use of Student’s *t*-test were not met or when variables were measured on an ordinal scale). Statistically significant differences between the groups were analyzed with post hoc tests (the Tukey test for the F test; and Dunn multiple comparisons for the Kruskal–Wallis test). For the model of two related variables, Student’s *t*-test or the paired samples Wilcoxon test were used (when conditions for the use of Student’s *t*-test were not met or when variables were measured on the ordinal scale). The significance of differences between more than two related variables in the model was verified by the analysis of variance with repeated measures or Friedman’s test (when conditions for the use of the analysis of variance with repeated measures or variables measured on the ordinal scale were not met). Chi-square independence tests were used for qualitative variables (Yate’s correction for cell numbers below 10, conditions for Cochran’s theorem, or the exact Fisher test). To establish the power and type of relationships between variables, we used correlation analysis by calculating Pearson and (or) Spearman correlation coefficients. In all calculations, the level of significance was adopted at *p* = 0.05. The Mann–Whitney–Wilcoxon (M-W-W) two-sided test with Bonferroni correction was used for statistical calculations with *p*-value annotation legend in figures characterized as:

- ns: 0.05 < *p* <= 1.00;
*: 0.01 < *p* <= 0.05;
**: 0.001 < *p* <= 0.01;
***: 0.0001 < *p* <= 0.001;
****: *p* <= 0.0001 .

## 3. Results

### 3.1. Study Group Characteristics

In the examined group of 112 patients, women were, on average, older than men, *p* = 0.0158. A total of 82 patients (73.2%) were overweight. The mean BMI for overweight people was 30.74 ± 3.56, while for those with normal weight, it was 22.85 ± 1.99. The basic characteristics of the study group in terms of age, BMI, and WHR are presented in Table 2.

The basic characteristics of the examined group in terms of total cholesterol (mg/dL), HDL, LDL, triglycerides (mg/dL), and LVEF/EF are presented in Table 3.

**Figure 1:**
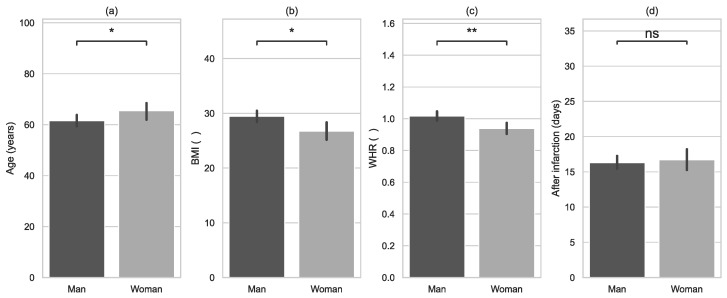
Characteristics of examined groups (sex) with error bars and *p*-value annotation legends: (**a**) age, (**b**) body mass index (BMI), (**c**) waist-to-hip ratio (WHR), (**d**) number of days after infarction and onset of rehabilitation. *: 0.01 < *p* <= 0.05; **: 0.001 < *p* <= 0.01.

The BMI was higher in men (Figure 1). The waist-to-hip ratio (WHR) was higher also in the men than in the women. However, there was no statistically significant difference between the groups in terms of the number of days after infarction and onset of rehabilitation.

**Figure 2:**
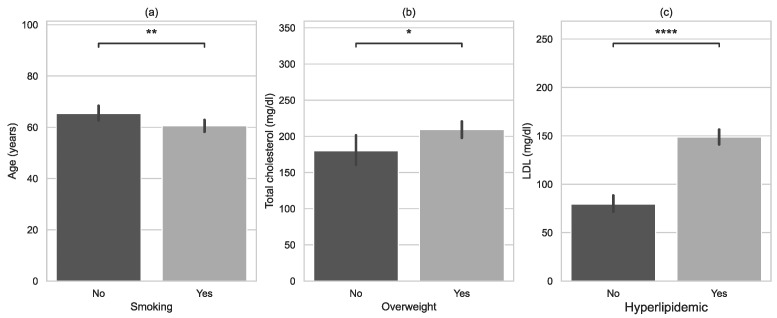
Characteristics of examined groups with error bars and *p*-value annotation legends: (**a**) age vs. smoking, (**b**) total cholesterol vs. overweight, (**c**) LDL vs. hyperlipidaemia. *: 0.01 < *p* <= 0.05; **: 0.001 < *p* <= 0.01; ****: *p* <= 0.0001.

In the study group, smokers were younger than non-smokers (Figure 2). Overweight patients had higher total cholesterol levels than normal-weight individuals. Hyperlipidaemia had a significant effect on low-density lipoprotein (LDL). Patients with hyperlipidaemia had significantly higher LDL levels. However, there was no statistically significant difference in the level of triglycerides between diabetic and non-diabetic subjects, where the *p*-value of the M-W-W test was 0.061.

**Figure 3:**
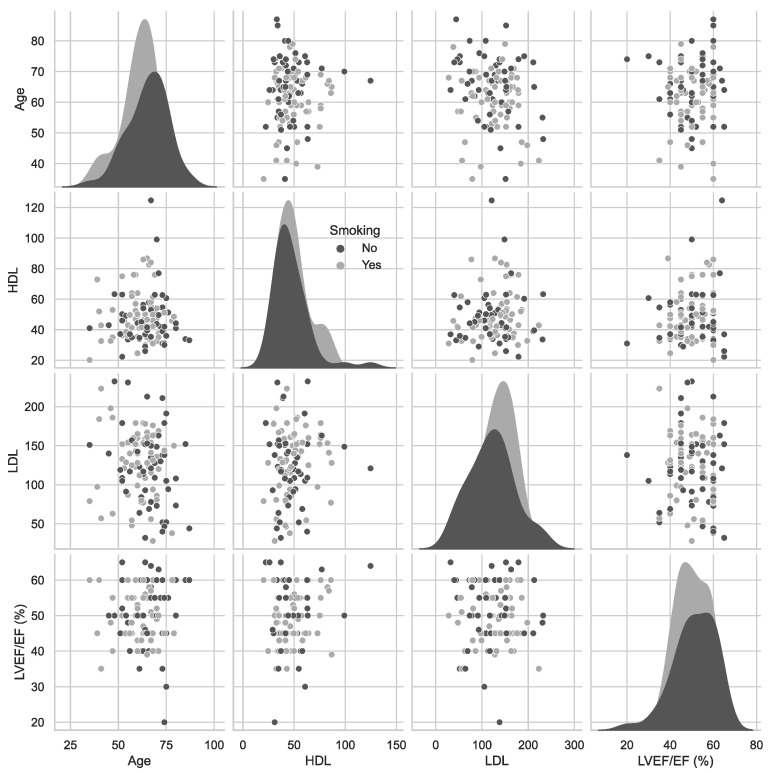
A pairwise relationship between variables age, HDL, LDL, and LVEF/EF.

Figure 3 presents a matrix of scatterplot that shows the pairwise relationship between variables such as age, HDL, LDL, and LVEF/EF in two groups (smoking: yes or no).

### 3.2. Risk Factors of Myocardiac Infarction

Initially, parameters measured before and after cardiac rehabilitation were compared for the total study group. Results are presented in Table 3. There was no significant difference between the resting heart rate (HR rest) measured before and after CR. However, an increase in the mean maximal heart rate (HR max) was observed, from 113.3 before CR to 122.9 after CR. Differences between other parameters were also significant. The final HR 1 min post-exercise, MET, DPr, and 6MWT were significantly higher after CR than before CR. Only the Borg rating of perceived exertion after the final exercise test was lower after CR than before CR.

We further analyzed the relationship between risk factors and parameters measured before and after CR. Table 4 and Table 5 present the results of statistical analysis for the relationship between specific variables. Significant differences are marked in red.

Overweight had the strongest influence on the increased value of initial: HR rest, HR max, and HR 1 min after exercise compared to subjects with normal BMI. DPr values before and after CR were also higher in overweight patients. Values of 6MWT were higher in smokers than in non-smokers. Diabetes influenced the final MET value. The final MET values were higher in non-diabetic subjects. Hyperlipidaemia was associated with a higher initial HR max and initial HR 1 min after exercise. DPr before rehabilitation was also higher. The initial and final MET values were lower in hypertensive patients. The Borg rating of perceived exertion measured after the final exercise test was also higher in hypertensive patients. Hypertension influenced the initial and final scores in the 6MWT, which were significantly higher in normotensive patients.

### 3.3. Correlation between Variables

**Figure 4:**
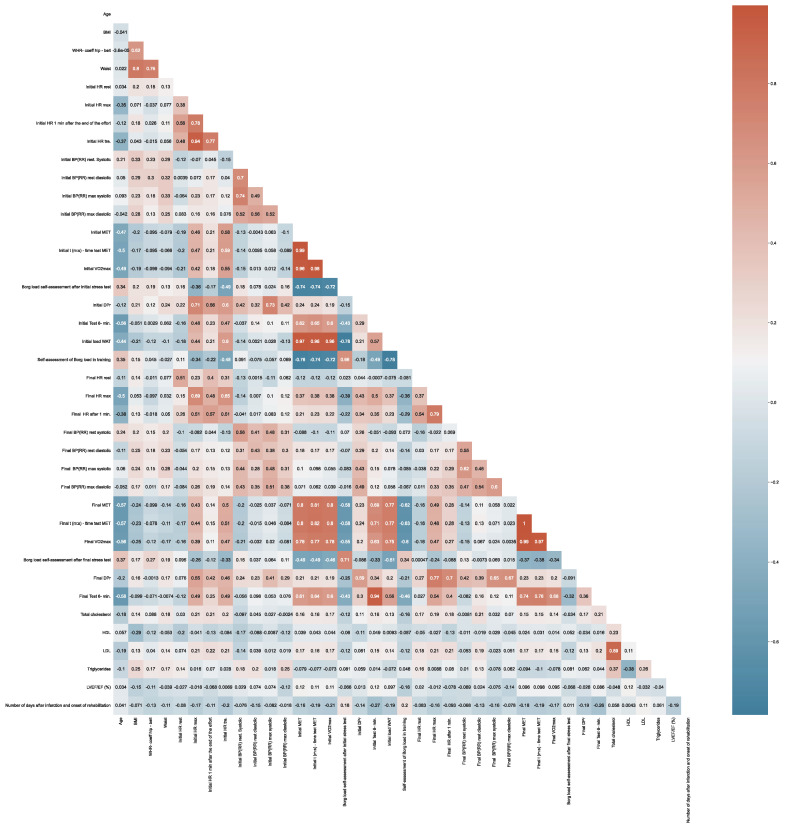
Correlation matrix between variables.

Figure 4 presents the correlation between variables. The correlation between two variables indicates that as one variable changes in value, the other variable tends to change in a specific direction. Figure 4 shows the relationships between particular variables, their strength, and direction.

The analysis revealed that increased values of waist correlated with increased BMI (*p* = 0.8). We also found a correlation between high levels of LDL and high levels of TC (*p* = 0.89). There was a significant positive correlation between the initial 6MWT and the final 6MWT (*p* = 0.94). There was a significant positive correlation between the final MET and initial MET *(p* = 0.8). We found a significant negative correlation between the Borg rating of perceived exertion and initial MET (*p* = −0.76) (Figure 4).

## 4. Discussion

The presented study assessed the impact of early cardiac rehabilitation under CSC-Infarct on the improvement in health in patients after myocardial infarction. Our findings indicated that the CSC-Infarct can improve symptoms, functional capacity, and health-related quality of life in MI patients. These benefits are associated with faster recovery in MI patients compared to groups that enter the second stage of CR later than the average of 10 days after MI [29].

The improved exercise capacity is reflected, among other things, in a decreased resting HR. It is true that in the analyzed group of patients, no significant changes in this parameter were found after CR was provided under the CSC-Infarct. This may be due to the short follow-up period (4 weeks). However, other researchers reported that CR significantly decreased HR rest [31]. The consequence of improved exercise tolerance after CR was a significant increase in the HR max in the study group. Contrary to our findings, some researchers did not report any significant increase in HR max after CR [31]. In the current study, we found a significant increase in the HR 1 min post-exercise measured after completed CR. Similar changes in HR measured 1 min after exercise were also observed by other authors [31,32].

We also found increased energy expenditure expressed in MET after CR compared to initial parameters measured before CR, supporting enhanced cardiorespiratory fitness. In the study group, a significant increase in METs (6.2 ± 2.0 to 8.4 ± 2.6) was found 4 weeks after CR under the CSC-Infarct. Similar observations have been made in other studies [31,33].

The DPr index provides information on the cardiovascular response at rest and during exercise. It is a very useful tool in cardiology to assess the severity of coronary heart disease [34]. DPr shows how the heart is coping with a given exercise and how much work it took to overcome the given load on a treadmill. The DPr may also be of great prognostic value in the assessment of cardiovascular function in healthy people with different fitness levels [29]. Our research revealed that the resting index based on DPr increased after the 4-weeks CSC-Infarct rehabilitation program. This is the only parameter that may indicate a deterioration of functional capacity in the studied population. However, it should be noted that the MET increased significantly, so the heart had to work harder, and thus the DPr parameter could have increased. The time from the initial to the final exercise test was short (4 weeks). Rehabilitation prolonged to at least 3 months could result in an increase in MET and a simultaneous reduction in DPr. Nevertheless, changes in other analyzed parameters have indicated an improvement in exercise capacity [35,36].

A significant increase in the 6MWT was observed after CR (477.5 ± 106.7 to 531.2 ± 98.1 min). Similar findings were also reported by other researchers [37].

In the presented study, decreased ratings on the Borg scale were observed after cardiac rehabilitation, which proves that rehabilitation reduced the perceived exertion after exercise in examined subjects.

Our study also analyzed the influence of risk factors on the parameters measured before and after cardiac rehabilitation. For instance, the effect of overweight on HR, MET, DPr, and 6MWT was assessed. Overweight influenced the values of HR rest, HR max, and HR 1 min after the test in comparison to subjects who had normal body weight on admission to the CR department (before rehabilitation). The values of resting DPr before and after CR were also higher in overweight patients compared to patients with normal body weight. In the post-CR tests performed under the CSC-Infarct, obese patients showed improvement in most of the analyzed parameters, although the improvement in exercise capacity was greater in non-obese patients. Overweight (measured by BMI) is associated with an increased risk of recurrent coronary events after MI, especially in obese subjects [38]. Dharmapria et al. [39] investigated a population of patients in which 33% were obese and 67% were non-obese. A statistically significant improvement in 6MWT was found in both groups (*p* < 0.0001). There was a significant improvement (*p* < 0.05) in 6MWT in non-obese subjects compared to obese subjects. However, non-obese subjects showed a better improvement in cardiovascular capacity compared to obese subjects after CR. Jayawardena et al. described their experimental study on dietary intervention in cardiac rehabilitation after MI. During the 12 weeks follow-up, a significantly higher mean weight loss was observed (intervention group: −1.27 ± 3.58 kg; control group: −0.26 ± 2.42 kg) in subjects from the intervention group compared to controls (*p* = 0.029). Moreover, the intervention group showed an insignificant reduction in blood pressure and blood lipid levels [40].

Smoking is an important modifiable risk factor for cardiovascular disease, causing approximately one in four deaths related to cardiovascular disease globally. In our study, there were no significant differences between smokers and non-smokers with respect to HR rest, HR max, HR 1 min, MET, Borg rating, or DPr. Our study revealed that 6MWT scores in smokers were significantly higher compared to non-smokers, both before and after CR. This may be due to the lower endurance of effort in smokers [41]. A review and meta-analysis of 18 studies (12 articles) [42] demonstrated that 53% of smoking patients with cardiovascular diseases quit smoking after participating in the CR program, which indicated that CR was effective in eliminating tobacco exposure. According to another analysis [43], 58% of the beneficial effects of CR can be attributed to the modification of cardiovascular risk factors, and about half of the 28% reduction in mortality related to cardiovascular disease can be attributed to a reduction in major risk factors, especially smoking. The study also found that after CR, more than half of patients with cardiovascular disease quit smoking. Smoking and obesity also reduce the health-related quality of life and increase the risk of further coronary events. Therefore, smoking cessation and adherence to dietary recommendations may be crucial in reducing mortality in all MI patients [41].

In our study, diabetes had no significant influence on HR rest, HR max, HR 1 min, Borg rating, DPr, or 6MWT both before CR and 4 weeks after it. Diabetes in patients after MI had a significant effect on the MET value after completing CR. The final MET values were higher in non-diabetic subjects.

A study by Laddu et al. [44] revealed less pronounced improvement in metabolic parameters in diabetic patients after the completion of CR, including abdominal obesity and lipid profiles (all *p* ≤ 0.002), compared to non-diabetic patients. As in our study, similar improvement was found in peak METs (*p* < 0.001) for both groups, but MET levels measured 12 weeks later remained lower in diabetic patients compared to non-diabetic patients.

In our study, for participants with hyperlipidaemia, the HR max, HR 1 min, and DPr before CR were higher, but after CR, there were no significant differences between the hyperlipidaemic and non-hyperlipidaemic groups. There were no significant differences in the HR rest, MET, Borg rating, or 6MWT before and after CR in the study group.

In the present study, patients with hypertension had significantly lower MET values and a significantly higher value of 6MWT before and after CR compared to normotensive patients. The Borg rating of perceived exertion was also significantly higher in hypertensive patients after CR compared to normotensive patients. No significant differences were found in this group for the HR rest, HR 1 min, Borg rating before CR, or DPr.

Before entering cardiac rehabilitation under CSC-Infarct, patients were also tested for levels of total cholesterol (201.6 ± 54.2), HDL (48.3 ± 16.6), LDL (25.3 ± 45.3), and triglycerides (167.3 ± 105.9). The LVEF/EF value, reflecting myocardial function, measured in study subjects on enrolment in CR under CSC-Infarct was 50.5 ± 8.4, which indicates a limit of the normal range or a moderate reduction in cardiac contractility [45]. We also assessed BMI and WHR, which were higher in men than in women. These parameters are associated with the lower exercise capacity and exercise tolerance of patients. The consequence of this is less effective work of the cardiovascular system and lower performance in exercise tests, as well as lower oxygen supply to the heart muscle.

Importantly, in addition to using CR up to 14 days after MI, long-term care beyond 4 weeks is required. Combining traditional care with the use of mobile technology offers the possibility of secondary prevention in high-level MI patients for an extended period of at least one year after MI. Research shows the effectiveness of such an action [46,47,48].

### Strengths and Limitations

The present study analyzed the influence of CR provided in Poland under CSC-Infarct in a group of patients after MI. The strength of this study is that it assessed the improvement in patients’ exercise capacity and exercise tolerance after a short 4-weeks rehabilitation and the influence of risk factors on this improvement. In addition, we used a multi-dimensional approach, accounting for many variables, which increased the reliability of statistical inference.

Nevertheless, the study also had some limitations. First, no control group was considered in the study design, and therefore we are unable to make conclusions on the causality of CR. Most patients from the analyzed population improved their exercise capacity during rehabilitation, but a similar improvement in patients not entering CR cannot be ruled out. However, this seems rather unlikely if we consider a large number of studies, including case-control trials, evidencing the positive effects of physical activity and exercise [1]. Second, the analyzed data included descriptive predictors that did not take into account the patients’ motivation to engage in exercise. Future research should also look at psychosocial factors known to be important in lifestyle modification, which can be assumed as not only crucial during CR but critical for maintaining regular exercise after the completed rehabilitation. Third, the rehabilitation period in our study was short (4 weeks) and had a significant positive effect on the analyzed parameters, but it is worth following the patients after MI for longer than just 4 weeks. Additional research is needed to improve our understanding of the relationship between CR and long-term clinical prognosis. This is a single-center study with a limited sample size. Research should be extended to other centers using CSC-Infarct.

## 5. Conclusions

CR within CSC-infarction in patients after myocardial infarction improves exercise tolerance. Five exercise sessions per week for 4 weeks was sufficient to improve exercise tolerance. Exercise tolerance in post-MI patients with concomitant risk factors is lower compared to post-MI patients without risk factors.

## Figures and Tables

**Table 1:** Rehabilitation models depending on risk and functional capacity.

Model A—patients with low risk and good functional capacity (>7 MET)	- Continuous endurance training for 3–5 days/week, 60–90 min/session, intensity 60–80% of heart rate reserve or 50–70% of maximal exertion level - Resistance training 2–3 sessions/week, 2–3 series/session - Exercise to improve general fitness 5 days/week
Model A—patients with moderate risk and good or moderate functional capacity (>5 MET)	- Continuous or interval endurance training for 3–5 days/week, 45–60 min/session, intensity 50–60% of heart rate reserve or 50% of maximal exertion level - Resistance training 2–3 days/week, one series/session - Exercise to improve general fitness for 5 days/week
Model C—patients with moderate risk and low functional capacity (3–5 MET) or high risk but good functional capacity (>6 MET)	- Moderate risk patients—interval endurance training for 3–5 days/week, 45 min/session, intensity 40–50% of heart rate reserve or 40–50% of maximal exertion level - High-risk patients—a set of exercises to improve general fitness for 5 days/week
Model D—patients with moderate risk and very low functional capacity (<3 MET) or high risk and moderate, low, or very low functional capacity (<6 MET)	- Personalized exercise 2–3 sessions/day for 3–5 days/week, 30–45 min/session, intensity under 20% of heart rate reserve or under a 10–15% increase in HR rest

**Table 2:** Characteristics of the study group.

		Study Group, Total N = 112	Women n = 34 (30.4%)	Men n = 78 (69.6%)	*p*-Value
Age	Mean (SD) Range Me 95% CI	62.8 (10.1) 35.0–87.0 64.0 [60.9;64.7]	65.5 (9.8) 39.0–80.0 67.0 [62.1;68.9]	61.6 (10.1) 35.0–87.0 62.5 [59.3;63.9]	0.0158 ^1^
BMI	Mean (SD) Range Me 95% CI	28.6 (4.8) 17.3–40.6 28.7 [27.7;29.5]	26.8 (4.6) 17.3–36.6 27.0 [25.1;28.4]	29.4 (4.6) 20.0–40.6 29.0 [28.4;29.0]	0.0054 ^2^
WHR	Mean (SD) Range Me 95% CI	0.99 (0.13) 0.65–1.4 0.98 [0.97;1.02]	0.94 (0.10) 0.65–1.2 0.92 [0.90;0.97]	1.01 (0.13) 0.8–1.4 1.0 [0.99;1.0]	0.0016 ^1^

^1^ U-M-W test, ^2^ *t*-Student test.

**Table 3:** Characteristics of the study group in terms of total cholesterol (mg/dL), HDL, LDL, triglycerides (mg/dL), and LVEF/EF.

Parameter	Total Cholesterol	HDL	LDL	Triglycerides	LVEF/EF
Mean (SD) Range Me	201.6 (54.2) 73.0–310.0 206.0	48.3 (16.6) 20.2–124.7 44.8	125.3 (45.3) 28.0–232.4 128.0	167.3 (105.9) 43.0–651.0 140.5	50.5 (8.4) 20.0–65.0 50.0
95% CI	[191.5;211.8]	[45.2;51.4]	[116.8;133.8]	[147.4;187.1]	[49.0;52.1]

HDL*—*high-density lipoprotein (mg/dL); LDL*—*low-density lipoprotein (mg/dl); LVEF/EF*—*left ventricular ejection fraction/ejection fraction (%).

**Table 4:** Comparative characteristics of the examined CSC-Infarct group in terms of: HR rest (min^−1^), HR max (min^−1^), HR 1 min (min^−1^), MET (mL *min^−1^ * kg^−1^), Borg perceived exertion, DPr, and 6MWT.

Rehabilitation	Parameter	HR Rest	HR Max	HR 1 Min	MET
Before rehabilitation	Mean (SD) Range Me 95% CI	72.4 (11.8) 47.0–117.0 71.0 [70.2;74.6]	113.3 (15.3) 73.0–158.0 114.5 [110.4;116.1]	90.4 (13.5) 55.0–143.0 90.0 [87.9;93.0]	6.2 (2.0) 3.0–10.1 6.3 [5.8;6.6]
After rehabilitation	Mean (SD) Range Me 95% CI	72.0 (10.8) 52.0–109.0 70.5 [70.0;74.1]	122.9 (17.0) 80.0–157.0 124.0 [119.7;126.1]	97.7 (14.1) 64.0–135.0 98.0 [95.1;100.3]	8.4 (2.6) 3.5–17.2 8.3 [7.9;8.9]
*p*-value		0.7174 ^1^	0.0000 ^1^	0.0000 ^1^	0.0000 ^2^
**Rehabilitation**	**Parameter**	**Borg RPE**	**DPr**	**6MWT**	
Before rehabilitation	Mean (SD) Range Me 95% CI	14.5 (0.6) 13.0–15.0 15.0 [14.4;14.6]	17,213.8 (3751.9) 8600.0–29,900.0 17,230.0 [16,511.3;17,916.4]	477.5 (106.7) 90.0–690.0 480.0 [457.5;497.5]	
After rehabilitation	Mean (SD) Range Me 95% CI	13.4 (0.7) 12.0–15.0 14.0 [13.3;13.5]	19,477.7 (4345.5) 10,400.0–31,120.0 19,355.0 [18,664.0;20,291.3]	531.2 (98.1) 240.0–750.0 540.0 [512.8;549.6]	
*p*-value		0.0000 ^2^	0.0000 ^2^	0.0000 ^2^	

Borg RPE—Borg rating of perceived exertion ^1^ *t*-Student test, ^2^ Wilcoxon test.

**Table 5:** The *p*-value parameters HR rest, HR max, HR 1 min, MET, DPr, and 6MWT between the examination before and after cardiac rehabilitation.

Variables	Overweight (No—Yes)	Smoking (No—Yes)	Diabetes (No—Yes)	Hyperlipidaemia (No—Yes)	Hypertension (No—Yes)
Initial HR rest	0.0394 ^1^	0.9278 ^1^	0.2882 ^1^	0.4836 ^1^	0.1060 ^1^
Final HR rest	0.1096 ^1^	0.9534 ^1^	0.3413 ^1^	0.2990 ^1^	0.8493 ^1^
Initial HR max	0.0394 ^1^	0.2403 ^1^	0.7934 ^1^	0.0423 ^1^	0.3793 ^1^
Final HR max	0.0593 ^1^	0.2133 ^1^	0.8603 ^2^	0.1868 ^2^	0.1543 ^2^
Initial HR 1 min	0.0046 ^1^	0.8839 ^1^	0.2908 ^1^	0.0032 ^1^	0.3166 ^1^
Final HR 1 min	0.0415 ^2^	0.1332 ^1^	0.6578 ^2^	0.5187 ^2^	0.6113 ^2^
Initial MET	0.4040 ^1^	0.3080 ^1^	0.1332 ^1^	0.4155 ^1^	0.0405 ^1^
Final MET	0.4698 ^1^	0.1088 ^1^	0.0272 ^1^	0.5677 ^1^	0.0154 ^1^
Initial Borg	0.3894 ^1^	0.7949 ^1^	0.1807 ^1^	0.7423 ^1^	0.0579 ^1^
Final Borg	0.3840 ^1^	0.8425 ^1^	0.6457 ^1^	0.8034 ^1^	0.0338 ^1^
Initial DPr	0.0005 ^1^	0.4129 ^2^	0.5258 ^1^	0.0332 ^1^	0.0541 ^1^
Final DPr	0.0043 ^2^	0.1270 ^3^	0.7355 ^2^	0.0809 ^2^	0.7301 ^2^
Initial 6MWT	0.4362 ^1^	0.0029 ^1^	0.3154 ^1^	0.4836 ^1^	0.0462 ^1^
Final 6MWT	0.4861 ^1^	0.0035 ^1^	0.3639 ^1^	0.3913 ^1^	0.0398 ^1^

^1^ U-M-W test, ^2^ t-Student test, ^3^ *t*-test with independent estimation (Welch).

## Data Availability

DAS is available on demand from corresponding author.
